# Notification that new names of prokaryotes, new combinations and new taxonomic opinions have appeared in volume 75, part 12 of the IJSEM

**DOI:** 10.1099/ijsem.0.007051

**Published:** 2026-03-31

**Authors:** Aharon Oren, Markus Göker

**Affiliations:** 1 The Institute of Life Sciences, The Hebrew University of Jerusalem, The Edmond J. Safra Campus, 9190401 Jerusalem, Israel; 2 Leibniz Institute DSMZ – German Collection of Microorganisms and Cell Cultures, Inhoffenstrasse 7B, 38124 Braunschweig, Germany

This listing of names of prokaryotes published in a previous issue of the *International Journal of Systematic and Evolutionary Microbiology* (*IJSEM)* is provided as a service to microbiology to assist in the recognition of new names and new combinations. This procedure was proposed by the Judicial Commission [1]. The names given herein are listed according to the Rules of priority (i.e. time and date of publication of the articles on the journal’s platform and the order of valid publication of names in the original articles) [2].

**Table:** 

Order of article publication	Name/authors	Proposed as	Article no.	Page no.
1	*Dryocola mayonis* Mohammed *et al*. 2025	sp. nov.	006986	8
1	*Dryocola sharpae* Mohammed *et al*. 2025	sp. nov.	006986	9
1	*Dryocola baronae* Mohammed *et al*. 2025	sp. nov.	006986	9
2	*Couchioplanes azureus* (Tamura *et al*. 1994) Kaewkla *et al*. 2025	comb. nov. [basonym: *Couchioplanes caeruleus* subsp. *azureus* Tamura *et al*. 1994]	006939	23
2	*Actinoplanes oryzae* Kaewkla *et al*. 2025	sp. nov.	006939	23
3	*Agarivorans sediminis* Bae *et al*. 2025	sp. nov.	006988	6
4	*Microbacterium luticellae* Tian *et al*. 2025	sp. nov.	006991	10
4	*Microbacterium alcoholitolerans* Tian *et al*. 2025	sp. nov.	006991	10
4	*Microbacterium daqui* Tian *et al*. 2025	sp. nov.	006991	10
5	*Flavobacterium adiutor* Le *et al*. 2025	sp. nov.	006958	9
6	*Jiella marina* Li *et al*. 2025	sp. nov.	006970	9
7	*Flagellimonas ulvae* Won *et al*. 2025	sp. nov.	006996	8
7	*Flagellimonas rhodophyticola* Won *et al*. 2025	sp. nov.	006996	8
8	*Streptosporangium heterodermiicola* Somphong *et al*. 2025	sp. nov.	006992	9
9	*Gordonia aurantiaca* Kim *et al*. 2025	sp. nov.	006994	9
9	*Nocardioides secundeburneus* Kim *et al*. 2025	sp. nov.	006994	11
10	*Vibrio sabulosilitoris* Machii *et al*. 2025	sp. nov.	007000	8
11	*Anaerozeibacter* El Houari *et al*. 2025	gen. nov.	006985	10
11	*Anaerozeibacter quisquiliarum* El Houari *et al*. 2025	sp. nov.	006985	12
11	*Anaerozeibacteraceae* El Houari *et al*. 2025	fam. nov.	006985	12
13	*Thermorhabdus* Xu *et al*. 2025	gen. nov.	006997	6
13	*Thermorhabdus hydrogeniformans* Xu *et al*. 2025	sp. nov.	006997	7
14	*Nocardioides proteolyticus* Liu *et al*. 2025	sp. nov.	007006	7
14	*Nocardioides ureilyticus* Liu *et al*. 2025	sp. nov.	007006	7
15	*Furfurilactobacillus cerevisiae* Zipori *et al*. 2025	sp. nov.	007009	6
15	*Furfurilactobacillus cerealis* Zipori *et al*. 2025	sp. nov.	007009	7
16	*Croceiramulus* Yoo *et al*. 2025*	gen. nov.	006995	8
16	*Croceiramulus getboli* Yoo *et al*. 2025	sp. nov.	006995	8
17	*Caproiciproducens puteiluti* Jin *et al*. 2025	sp. nov.	007008	8
17	*Caproiciproducens lactatisolvens* Jin *et al*. 2025	sp. nov.	007008	8
18	*Methylomonas stagni* Gwak *et al*. 2025	sp. nov.	007002	10
19	*Maribacter algae* Kim *et al*. 2025	sp. nov.	006993	9
19	*Maribacter spongiae* Kim *et al*. 2025	sp. nov.	006993	10
19	*Maribacter corallii* Kim *et al*. 2025	sp. nov.	006993	10
19	*Maribacter aquimarinus* Kim *et al*. 2025	sp. nov.	006993	10
19	*Maribacter taeanensis* Kim *et al*. 2025	sp. nov.	006993	11

*The protologue must give *Croceiramulus getboli* instead of *C. getboli* as the type species.

The following taxonomic opinions (i.e. the emendation of circumscriptions or the creation of synonyms) do not affect the status of a name under the International Code of Nomenclature of Prokaryotes. These taxonomic opinions can neither be considered as approved nor as disapproved by the International Committee on Systematics of Prokaryotes.

**Table:** 

Name/authors	Article no.	Page no.
*Dryocola* Maddock *et al*. 2023 emend. Mohammed *et al*. 2025	006986	9
*Fontibacillus solani* Ramírez-Bahena *et al*. 2015 pro synon. *Fontibacillus panacisegetis* Lee *et al*. 2011	007007	8
*Geobacter anodireducens* Sun *et al*. 2014 emend. Dif *et al*. 2025	007005	9
*Geobacter soli* Zhou *et al*. 2014 pro synon. *Geobacter anodireducens* Sun *et al*. 2014	007005	8
*Metallosphaera prunae* Fuchs *et al*. 1996 pro synon. *Metallosphaera sedula* Huber *et al*. 1989	007004	6
*Peribacillus castrilensis* Rodríguez *et al*. 2023 pro synon. *Peribacillus frigoritolerans* (Delaporte and Sasson 1967) Montecillo and Bae 2022	006998	3
*Peribacillus frigoritolerans* (Delaporte and Sasson 1967) Montecillo and Bae 2022 emend. McLaughlin *et al*. 2025	006998	3
*Virgibacillus kapii* Daroonpunt *et al*. 2016 pro synon. *Virgibacillus salexigens* (Garabito *et al*. 1997) Heyrman *et al*. 2003	006990	9
*Virgibacillus massiliensis* Khelaifia *et al*. 2023 pro synon. *Virgibacillus salexigens* (Garabito *et al*. 1997) Heyrman *et al*. 2003	006990	9
*Virgibacillus salexigens* (Garabito *et al*. 1997) Heyrman *et al*. 2003 emend. Menasria and Zaatout 2025	006990	9
